# Impact of Induction Therapy in Low Immunological Risk Simultaneous Pancreas-Kidney Transplantation

**DOI:** 10.3389/ti.2025.15263

**Published:** 2025-09-30

**Authors:** Enrique Montagud-Marrahi, Adriana Rodriguez-Gonzalo, Joan Vidiella-Martin, Blanca Martin Álvarez, Irving Gaston Ramírez, Albert Baronet, Joana Ferrer-Fàbrega, Antonio J. Amor, Maria José Ramírez-Bajo, Mireia Musquera, Fritz Diekmann, Pedro Ventura-Aguiar

**Affiliations:** ^1^ Kidney Transplant Unit, Nephrology and Kidney Transplantation Department, Hospital Clinic of Barcelona, Barcelona, Spain; ^2^ Laboratori de Nefrologia i Trasplantament (LENIT), Fundació per a la Recerca Biomèdica - Institut d’Investigacions Biomèdiques August Pi i Sunyer, Barcelona, Spain; ^3^ Red de Investigación Cooperativa Orientada a Resultados en Salud (RICORS 2040), Madrid, Spain; ^4^ Nephrology Department, Consorci Corporació Sanitària Parc Taulí, Sabadell, Spain; ^5^ Nephrology Department, Hospital Universitario Río Hortega, Valladolid, Spain; ^6^ Nephrology Department, Centro Medico Nacional 20 de Noviembre, Mexico City, Mexico; ^7^ Hepatobiliopancreatic and Liver Transplant Department, Hospital Clínic Barcelona, Barcelona, Spain; ^8^ Diabetes Unit, Endocrinology and Nutrition Department, Hospital Clínic Barcelona, Barcelona, Spain; ^9^ Urology Department, Hospital Clinic de Barcelona, Barcelona, Spain

**Keywords:** simultaneous kidney pancreas transplantation, thymoglobulin, basiliximab, opportunistic infections, neoplasm

## Abstract

T-cell depleting agents and IL-2 receptor blockers are the most common induction therapies in simultaneous pancreas-kidney transplantation (SPKT), but the optimal choice remains debated. Here, we perform a retrospective, single-center study with SPKT recipients from 2000 to 2023. Basiliximab was used between 2008 and 2013, and thymoglobulin in other periods. Patients with prior transplants, calculated PRA >20%, pre-SPKT Donor-Specific Antibodies or graft primary non-function because technical reasons, were excluded. An Inverse Probability of Treatment Weighting (IPTW) was performed to adjust for confounding variables. 305 SPKT recipients were included, of which 172 (56%) received thymoglobulin and 133 (44%) basiliximab. Recipient (86% vs. 80%), pancreas (86% vs. 83%) and kidney (84% vs. 89%) death-censored graft survival at 20 years were comparable between groups. Basiliximab was not associated with an increased risk of patient death [HR 1.47 (0.69–3.14), P = 0.32], pancreas [HR 1.08 (0.55–2.10), P = 0.83] or kidney graft failure [HR 0.80 (0.38–1.70), P = 0.56] compared to thymoglobulin. Basiliximab did not significantly increase the risk of pancreas [OR 1.49 (0.84–2.63), P = 0.37] or kidney graft rejection [OR 1.31 (0.54–3.15), P = 0.20]. However, it was associated with significantly lower risk of CMV [OR 0.41 (0.23–0.72), P = 0.002] and BK virus infections [OR 0.31 (0.12–0.80), P = 0.02]. No significant difference was found in new-onset malignancy incidence. These results were maintained even after IPTW adjustment. In SPKT recipients with low immunological risk, basiliximab provides comparable long-term patient and graft outcomes to thymoglobulin while reducing the incidence of opportunistic infections.

## Graphical Abstract

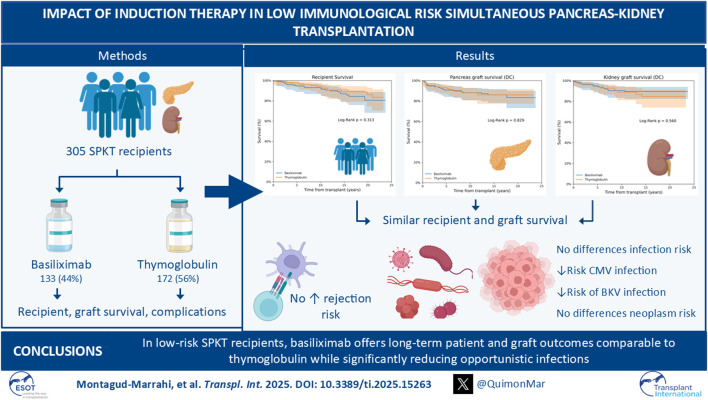

## Introduction

Simultaneous Pancreas-Kidney Transplantation (SPKT) has proven to be an effective therapy for patients with End-Stage Kidney Disease (ESKD) and insulin-dependent Diabetes Mellitus (DM), reducing the incidence of major cardiovascular events while improving patient survival and quality of life [1–5].

Despite significant advances in immunosuppressive therapy in recent years, allograft rejection remains one of the most common causes of graft loss, especially after 1 year post-transplant [5–7]. Immunosuppressive regimen for SPKT includes induction therapy, typically with either a T-cell depleting agent (e.g., thymoglobulin) or an IL-2 receptor blocker (e.g., basiliximab) administered in the immediate post-transplant period, followed by maintenance therapy, which usually consists of a combination of steroids, calcineurin inhibitors, and an antiproliferative agent (such as mycophenolate or mTOR inhibitors) [8]. Most transplant centers use T-cell depleting agents for SPKT as induction therapy, regardless of the recipient’s immunological risk prior to transplantation [9, 10]. However, evidence comparing SPKT outcomes between T-cell depleting agents and basiliximab is controversial, particularly in patients with low immunological risk [8–10]. Identifying the most appropriate induction therapy for SPKT recipients is increasingly important, as T-cell depleting agents have been linked to higher rates of opportunistic infections and *de novo* malignancies, negatively impacting on recipient survival [10–12].

In the present study we compare post-transplant outcomes in SPKT recipients receiving either thymoglobulin or basiliximab as induction therapy. Specifically, we analyze patient and graft survival, rejection rates, incidence of infections, and the occurrence of *de novo* malignancies following transplantation.

## Materials and Methods

### Study Design

We conducted a longitudinal retrospective single center study including all SPKT performed at Hospital Clínic Barcelona from January 1st, 2000 until December 31st, 2023 (n = 385). Patients with ≥1 previous transplant of any type (n = 19), pre-transplant calculated Panel Reactive Antibody (cPRA) >20% and/or Donor Specific Antibodies (DSAs) (n = 54), and those with a kidney or pancreas graft primary non-function for technical reasons (n = 7) were excluded. In total, 305 SPKT recipients were included.

According to our immunology laboratory, a bead in the Single Antigen assay was considered positive when the mean fluorescence intensity (MFI) was ≥1,000. However, this threshold was subject to minor patient-specific adjustments based on the background MFI observed in non–donor-specific beads, which could result in a slightly higher or lower effective cut-off.

Data was collected until 31st December 2024. The clinical and research activities being reported were consistent with the Declaration of Istanbul on Organ Trafficking and Transplant Tourism. The study protocol was approved by the Research Ethics Committee (HCB/2025/0613).

### Immunosuppression

Induction immunosuppression therapy was used in all patients, with two doses of basiliximab of 20 mg at day 0 and at day +4 after surgery between January 2008 until July 2013. Before January 2008, and after July 2013, rabbit anti-human lymphocytes polyclonal antibodies (either Thymoglobulin^®^ 1.25 mg/kg/day or ATG^®^ 2.5 mg/kg/day, for 4 consecutive days) was administered as induction therapy. The first dose was administered intraoperatively, and the subsequent three doses on consecutive days following surgery. Dosage was adjusted according to leukocyte and platelet counts: it was reduced by 50% if the leukocyte count was <3.000/mL and/or the platelet count was <75,000/mL. If the leukocyte count fell below 1.500/mL and/or the platelet count below 50.000/mL, the dose was postponed (not discontinued) until the following day to try to reach a total cumulative dose of 5 mg/kg (10 mg/kg for ATG).

Maintenance immunosuppression protocol was based on triple therapy with calcineurin inhibitor (cyclosporine A until 2005, and thereafter tacrolimus), mycophenolate or mTOR inhibitors, and steroids (methylprednisolone in the immediate post-transplant period, followed by oral prednisone). The first dose of the calcineurin inhibitor was administered immediately before surgery. Administration was not postponed in cases of kidney Delayed Graft Function (DGF), and dosage adjustments were made solely based on trough levels. Therefore, all patients received the same regimen regardless of kidney DGF occurrence and induction therapy.

### Anticoagulation

Anticoagulation included subcutaneous enoxaparin 20 mg bid starting 8 h post-surgery and was maintained until patient discharge (in the absence of thrombotic/hemorrhagic complications), and acetylsalicylic acid 50 mg/day starting at 12 h post-surgery until discharge, when it is increased up to 100 mg/day.

### Infections and *De Novo* Neoplasms

Infections were considered when requirement for hospital admission. Cytomegalovirus (CMV) prophylaxis with valganciclovir was administered to all patients for 1 month post-transplant or three if a donor/recipient mismatch for CMV was present. Infection was defined as any replication in CMV load post-transplant, regardless of the presence of CMV disease. BK nephropathy was defined an increase in BK viremia >10.000 UI/mL, regardless of the presence of biopsy proven BK nephropathy.

*De novo* neoplasms were considered as any neoplasia diagnosed during the post-transplant period, including solid tumours, post-transplant lymphoprolipherative disorders (PTLD) and excluding non-melanoma skin cancer.

### Outcomes

Primary outcomes were recipient survival and death-censored kidney or pancreas graft survival at 1, 5, 10, and 20 years after transplantation, and graft rejection during follow up. Our secondary outcomes were defined as number of infections requiring hospital admission, CMV infection, BK virus nephropathy, and new onset neoplasms.

Patient survival was calculated from the date of transplantation to the date of death from any cause. Patients alive at the last follow-up were censored at that date. Pancreas graft failure was defined as any of the following: a) graft removal, b) C-peptide <1 ng/mL or c) total daily insulin dose >0.5 U/Kg.

Kidney graft failure was defined as return to dialysis or re-transplantation. Kidney DGF was defined as the need for at least one session of hemodialysis during the first week following SPKT.

Graft survival was analyzed using death-censored estimates to evaluate the effect of induction therapy on graft failure, particularly from immunological causes, independent of patient mortality. Nevertheless, to reduce the risk of bias derived from potential competing risks between recipient death and graft failure, a competing risk analysis was also performed.

### Rejection Diagnosis and Treatment

All rejection episodes (for both pancreas and kidney) were biopsy-proven. Diagnostic criteria were based on the Banff classification in use at the time of diagnosis for either pancreas or kidney grafts. In cases of pancreas T cell–mediated rejection (TCMR), patients received three doses of methylprednisolone (500 mg/day) followed by five doses of thymoglobulin (1.25 mg/kg/day). For pancreas antibody-mediated rejection (ABMR), treatment consisted of three doses of methylprednisolone (250 mg/day), two doses of rituximab (400 mg/day), and five sessions of plasma exchange. For kidney graft rejection, the same therapeutic protocols were applied, except in cases of TCMR grade I, in which thymoglobulin was not administered.

### Statistical Analysis

Data are presented as mean (standard deviation, SD) for continuous variables and median [interquartile range (IQR)] for the non-continuous ones. The corresponding tests used were t-test, Mann-Whitney test, Chi-square or Fisher’s Exact test as appropriated. Competing risk analysis for graft survival was performed using the Fine–Gray subdistribution hazard model.

Inverse probability of treatment weighting (IPTW) was used to account for covariate imbalance between basiliximab and thymoglobulin groups. IPTW was estimated from a propensity score from a logistic regression model to receive basiliximab as the induction agent. The model included factors associated with the donor and either of the outcomes: dialysis duration before transplant, diabetes duration before transplant, time on the waiting list, HLA mismatches between donor and recipient, type of maintenance immunosuppression, prednisone withdrawal, recipient age at transplantation, cold ischemia time for kidney graft, Pancreas Donor Risk Index (PDRI), pancreas transplantation era, recipient smoking habit, cPRA before transplant.

A stabilized weighting method was performed by multiplying the IPTW by the proportion of recipients treated with basiliximab and thymoglobulin. Check for adequate balance of covariates after IPTW analyses was performed by calculation of standardized differences and an absolute difference greater than 0.2 represented a meaningful imbalance. All subsequent analyses were performed on the weighted, covariate-balanced population. Kaplan-Meier was used to estimate patient and graft survival and compared using a log-rank test. Logistic regression was used to calculate odds ratio for graft rejection, infections and neoplasms, and Cox proportional regression was performed to estimate patient and graft hazards.

All variables analyzed presented less than 10% of missing values. Given the low percentage, imputation methods were not applied, and analyses were conducted with the available data.

Statistical analysis was performed using IBM SPSS Statistics 30.0 (SPSS, Inc; Chicago, Illinois) software for MacOS and Python programming language (Python Software Foundation, 2024) in MacOS. All tests were two-tailed and a significance of 0.05 was used. Graphs were generated using the Python programming language in MacOS.

## Results

### Recipient and Donor Characteristics

A total number of 305 SPKT recipients were included in the study (Table 1). In 172 (56%), thymoglobulin was used as the induction agent, while in 133 (44%) basiliximab was administered as the induction therapy. The mean follow up time for the whole cohort was 12.08 ± 6.84 years. Recipient age at SPKT was similar between both groups. Type 1 Diabetes (T1D) was predominant in both groups, in which diabetes duration was also similar. Most of the patients were on dialysis at SPKT in both groups, although time on dialysis before transplant was higher in the basiliximab one, as well as time on the waiting list. Smoking habit was more frequent in the basiliximab group, as well as patients at high risk of CMV infection.

**TABLE 1:** Baseline characteristics of included recipients.

	Thymoglobulin (n = 172)	Basiliximab (n = 133)	P value
Gender (Male)	102 (59)	88 (66)	0.24
Ethnicity			0.59
Caucasian	162 (94)	128 (95)	
Hispanic	9 (5)	5 (5)	
Asian	1 (1)	0 (0)	
Age at SPKT (years)	40.56 ± 7.58	40.96 ± 7.19	0.64
BMI (kg/m^2^)	23.60 ± 5.30	22.90 ± 5.40	0.26
Diabetes Mellitus type			1.00
Type 1	171 (99)	132 (99)	
Type 2	0 (0)	0 (0)	
Other types	1 (1)	1 (1)	
Diabetes Mellitus duration at SPKT (years)	25 [21–31]	24 [20–31]	0.11
Dialysis before transplant	146 (85)	117 (88)	0.50
Dialysis duration (months)	23 [13–34]	31 [21–40]	<0.001
Waiting list duration at SPKT (months)	10.5 [4.75–18.25]	17 [9–27]	0.002
Retinopathy	168 (98)	126 (95)	0.22
Neuropathy	91 (53)	60 (45)	0.17
Ischemic Heart Disease	16 (10)	22 (17)	0.08
Peripheral Artery Disease	44 (26)	41 (31)	0.37
Hypertension	121 (70)	86 (65)	0.50
Smoking habit	57 (40)	72 (54)	0.04
Transplant era (after 2008)	120 (69)	82 (62)	0.15
High risk of CMV infection	13 (8)	26 (20)	0.003
Total HLA mismatches			0.38
0–2	2 (1)	0 (0)	
3–4	30 (17)	20 (15)	
≥5	140 (82)	113 (85)	
cPRA pre-transplant >5%	8 (5)	8 (6)	0.61
Maintenance immunosuppression			0.22
FK + MMF	164 (95)	129 (97)	
FK + mTORi	6 (4)	1 (1)	
CsA + MMF	2 (1)	3 (2)	
Prednisone withdrawal	46 (27)	44 (33)	0.31
Tacrolimus trough levels (ng/mL)			
1 month	10.86 ± 2.82	11.82 ± 1.46	0.49
6 months	9.66 ± 1.66	9.80 ± 0.92	0.44
12 months	9.14 ± 1.02	8.32 ± 1.28	0.27
5 years	7.52 ± 0.94	7.07 ± 0.53	0.37
Kidney DGF	13 (8)	17 (13)	0.17

Data are means ± SD, n (%) or median [IQR] unless otherwise indicated. SPKT, simultaneous pancreas-kidney transplantation; BMI, body mass index; CMV, cytomegalovirus; HLA, human leukocyte antigen; cPRA, calculated Panel Reactive Antibody; PDN, prednisone; FK, tacrolimus; MPS, mycophenolate; mTORi, mTOR, inhibitors; CsA, cyclosporine; DGF, delayed graft function.

Table 2 summarizes donor characteristics. Age at donation was similar between both groups. No differences were observed for PDRI score among the studied groups. Donors after Circulatory Death (DCD) were more frequent in the thymoglobulin group. Pancreas and kidney Cold Ischemia Time (CIT) were longer in the basiliximab one.

**TABLE 2:** Donor characteristics.

	Thymoglobulin (n = 172)	Basiliximab (n = 133)	P value
Gender (Male)	100 (60)	81 (61)	0.91
Age (years)	32.85 ± 12.22	32.43 ± 10.55	0.76
BMI (kg/m^2^)	23.72 ± 3.28	23.47 ± 2.94	0.54
Hypertension history	12 (8)	4 (3)	0.07
Smoking habit	40 (28)	34 (28)	1.00
Alcohol consumption	15 (10)	6 (5)	0.11
PDRI risk	1.35 ± 0.61	1.30 ± 0.38	0.55
ICU Length of Stay (days)	2 [1–4]	2 [1–5]	0.64
Donation after Circulatory Death	22 (15)	2 (2)	<0.001
Pancreas CIT (hours)	8.77 ± 2.54	11.19 ± 3.11	<0.001
Kidney CIT (hours)	10.93 ± 2.82	13.36 ± 3.23	<0.001

Data are means ± SD, n (%) or median [IQR] unless otherwise indicated. BMI, body mass index; PDRI, pancreas donor risk index; ICU, intensive care unit; CIT, cold ischemia time.

After IPTW adjustment, no significant differences were observed between both groups neither for recipient nor for donor characteristics. Supplementary Table S1 shows standardized differences for donor and recipient characteristics before and after IPTW adjustment.

### Recipient Survival

**FIGURE 1:**
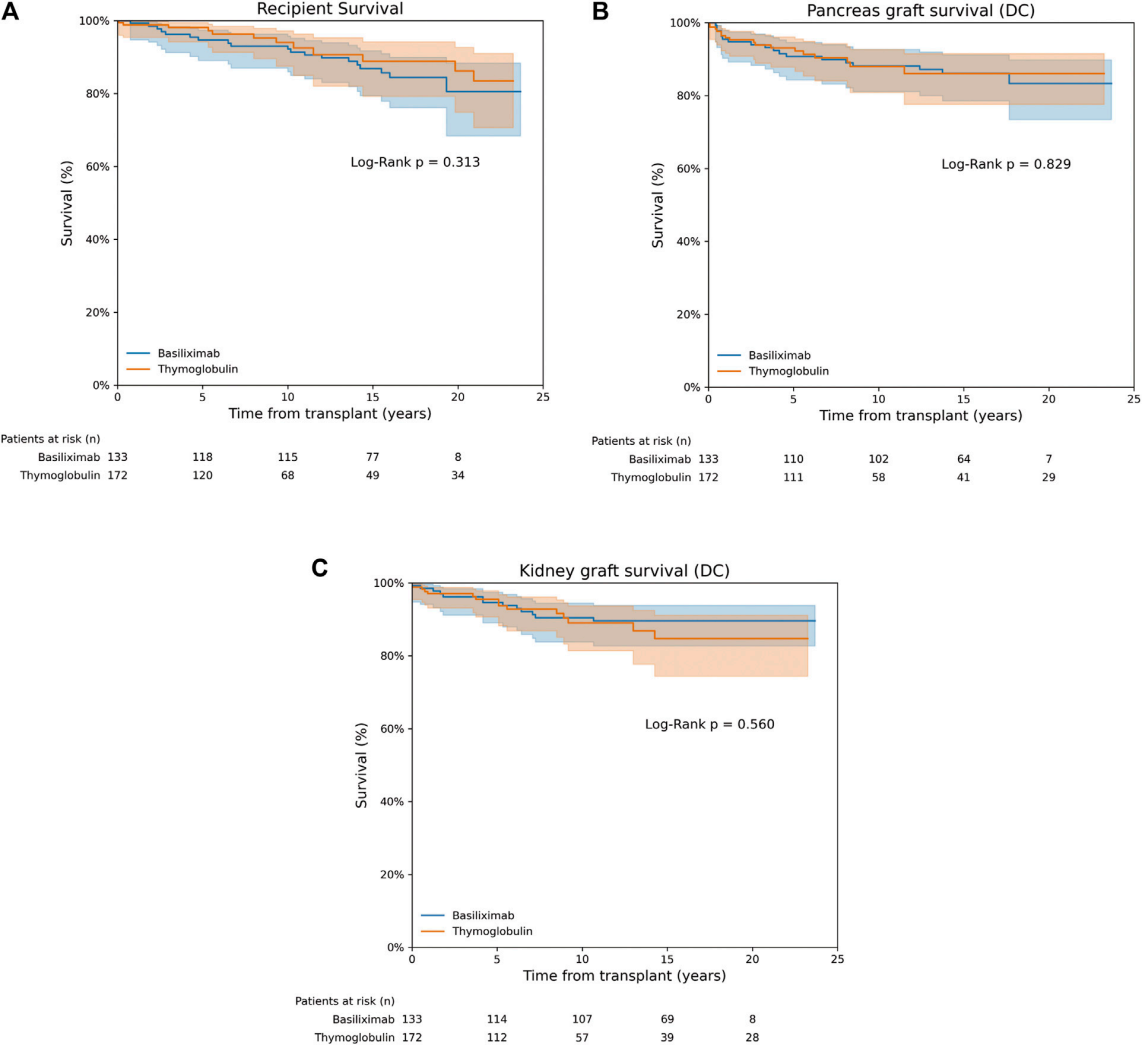
Recipient, pancreas and kidney graft survival. **(A)** Recipient survival. **(B)** Death-censored pancreas graft survival. **(C)** Death-censored kidney graft survival.

Patient survival at 1, 5, 10, and 20 years after SPKT was 98.8%, 98.1%, 94% and 86.2% in the thymoglobulin group, respectively. For basiliximab group, survival was not significantly different, being 99.2%, 94.6%, 92.2% and 80.5% for the same time periods, respectively (Log Rank P = 0.31) (Figure 1A). Unadjusted Cox regression analysis showed that basiliximab was not associated with an increased risk of patient death compared to thymoglobulin [HR 1.47 (0.69–3.14), P = 0.32]. A similar scenario was observed after IPTW adjustment, with no difference for patient death comparing both groups [HR 1.01 (0.43–2.34) for basiliximab group, P = 0.99] (Table 3). The main cause of recipient death in both groups were infections (50% vs. 37% for thymoglobulin and basiliximab groups, respectively), followed by neoplasms (34% vs. 26% for thymoglobulin and basiliximab, respectively), with no differences between groups (P = 0.55) (Supplementary Table S2).

**TABLE 3:** Non-adjusted and IPTW-weighted Cox regression for patient, pancreas and kidney graft survival.

	HR [95% CI]^a^	P value
Non-adjusted		
Patient death	1.47 [0.69–3.14]	0.32
Pancreas graft failure	1.08 [0.55–2.10]	0.83
Kidney graft failure	0.80 [0.38–1.70]	0.56
IPTW-weighted		
Patient death	1.01 [0.43–2.34]	0.99
Pancreas graft failure	1.57 [0.75–3.28]	0.24
Kidney graft failure	1.49 [0.84–2.63]	0.17

^a^ The Thymoglobulin group was considered the reference group.

### Pancreas Graft Survival

In the thymoglobulin group, pancreas death-censored graft survival at 1, 5, 10, and 20 years after SPKT was 95.9%, 93.1%, 88%, 86.1%, respectively. For basiliximab one, pancreas graft survival was similar, being 95.5%, 90.7%, 88.2%, 83.3% for the same time periods, respectively (Log Rank P = 0.83) (Figure 1B). No differences were observed for overall pancreas graft survival (Log Rank P = 0.57, Supplementary Figure S1A). Unadjusted Cox regression analysis showed no increased risk of pancreas failure with basiliximab compared to thymoglobulin as induction [HR 1.08 (0.55–2.10), P = 0.83]. These results were maintained after IPTW weighting [HR 1.57 (0.75–3.28) for basiliximab group, P = 0.24] (Table 3). A similar scenario was observed when performing a competing risk analysis, with a HR 0.93 [0.48–1.83], P = 0.84 for the basiliximab group.

### Kidney Graft Survival

Kidney graft survival rates in the thymoglobulin group at 1, 5, 10, and 20 years post-SPKT were 97.1%, 95.5%, 89%, and 84.7%, respectively. Similarly, kidney graft survival in the basiliximab group was 98.5%, 94.6%, 90.4%, and 89.6% at the corresponding time points (Log Rank P = 0.56) (Figure 1C). No differences were observed for overall kidney graft survival (Log Rank P = 0.99, Supplementary Figure S1B). According to unadjusted Cox regression analysis, there was no significant increase in the risk of kidney graft failure with basiliximab when compared to thymoglobulin [HR 0.80 (0.38–1.70), P = 0.56]. This finding remained consistent following IPTW adjustment [HR 1.49 (0.84–2.63) for the basiliximab group, P = 0.17] (Table 3). A similar scenario was observed when performing a competing risk analysis, with a HR 1.24 [0.57–2.70], P = 0.58 for the basiliximab group.

### Graft Rejection

Throughout the entire follow-up period, 34 pancreas rejection episodes (20%) occurred in the thymoglobulin group and 32 episodes (24%) in the basiliximab group, with no statistically significant difference between them (P = 0.40) (Table 4). Rejection occurred after a median time of 4 [1–23] and 6 [1–13] months for the thymoglobulin and basiliximab groups, respectively (P = 0.69).

**TABLE 4:** Pancreas and kidney graft rejection during follow up.

	Thymoglobulin (n = 172)	Basiliximab (n = 133)	P value
Pancreas rejection	34 (20)	32 (24)	0.40
ABMR	5 (3)	0 (0)	0.06
TCMR	29 (17)	32 (24)	
Kidney rejection	11 (7)	14 (11)	0.19
ABMR	5 (3)	2 (2)	0.10
TCMR	6 (4)	12 (9)	

Data are n (%). ABMR, Antibody-Mediated Rejection. TCMR, T Cell-Mediated Rejection.

In both groups, the most frequent type of pancreas rejection was TCMR, with 29 (17%) and 32 (24%) cases for thymoglobulin and basiliximab, groups, respectively. There was no statistical support for an association between rejection type and treatment group (P = 0.06). However, a tendency toward a different rejection pattern was observed for the pancreas graft: in the basiliximab group, all cases were TCMR, whereas in the thymoglobulin group, 3% of cases were ABMR. Pancreas graft rejection was the cause of graft loss in 14 cases in the thymoglobulin group and 11 cases in the basiliximab group, representing 82% and 61% of all graft losses (41% and 34% of treatment failure), respectively (P = 0.16).

Kidney graft rejection rate was similar between thymoglobulin and basiliximab groups, observing 11 (7%) and 14 (11%) kidney graft rejection episodes (P = 0.19). Rejection occurred after a median time of 8 [1–53] and 8 [2–44] months for the thymoglobulin and basiliximab groups, respectively (P = 0.93).

The most frequent type in both groups was TCMR (4% and 9% in the thymoglobulin and basiliximab groups, respectively). No statistically significant association was found between rejection type and treatment group (P = 0.10) (Table 4). Kidney graft rejection was the cause of graft loss in 2 cases in the thymoglobulin group and 1 case in the basiliximab group, representing 13% and 8% of all graft losses (18% and 7% of treatment failure), respectively (P = 0.63).

When assessing the risk of graft rejection, basiliximab was not associated with a higher risk of rejection, either for pancreas [OR 1.28 (0.74–2.22), P = 0.37] or kidney graft [OR 1.72 (0.76–3.93), P = 0.20] compared to thymoglobulin. These results were consistent after IPTW weighting, with an OR of 1.49 [0.84–2.63] (P = 0.17) for pancreas and 1.31 [0.54–3.15] (P = 0.55) for kidney graft and basiliximab compared to thymoglobulin (Table 5).

**TABLE 5:** Non-adjusted and IPTW-weighted logistic regression for pancreas and kidney graft rejection.

	OR [95% CI]^a^	P value
Non-adjusted		
Pancreas graft rejection	1.28 [0.74–2.22]	0.37
Kidney graft rejection	1.72 [0.76–3.93]	0.20
IPTW-weighted		
Pancreas graft rejection	1.49 [0.84–2.63]	0.17
Kidney graft rejection	1.31 [0.54–3.15]	0.55

^a^ The Thymoglobulin group was considered the reference group.

### Infections and New Onset Neoplasms

The rate of post-transplant infections (except for CMV and BKV) that required patient admission was similar between thymoglobulin and basiliximab (35% vs. 38%, respectively. P = 0.55). Infections occurred after a median time of 35 [1–109] and 34 [22–80] days for thymoglobulin and basiliximab, respectively (P = 0.57) Nevertheless, when specifically considering CMV infection and BK nephropathy, thymoglobulin group exhibited a significantly higher rate of CMV infection (29% vs. 14% for thymoglobulin vs. basiliximab, respectively. P = 0.002) and BK nephropathy (12% vs. 4%, P = 0.01) (Table 6). CMV infection occurred after a median time of 3 [2–5] and 3 [1–5] months (P = 0.42), while BK infection occurred after a median time of 9 [6–30] and 28 [23–30] months for thymoglobulin and basiliximab, respectively (P = 0.40). Induction with basiliximab was significantly associated with a reduced risk of CMV infection [OR 0.39 (0.21–0.70), P = 0.002] and BK nephropathy (OR 0.28 [0.10–0.77], P = 0.01) compared to thymoglobulin (Table 7). This association was maintained after IPTW adjustment (OR 0.41 [0.23–0.72], P = 0.002 for basiliximab and CMV infection; OR 0.31 [0.12–0.80], P = 0.02 for basiliximab and BK infection).

**TABLE 6:** Infections and new onset neoplasms during follow up.

	Thymoglobulin (n = 172)	Basiliximab (n = 133)
Infections that require hospitalization	59 (35)	51 (38)
CMV infection	48 (29)	18 (14)
BK infection	21 (12)	5 (4)
New onset neoplasms	11 (6)	14 (10)
PTLD	2 (1)	3 (2)
Breast	2 (1)	0 (0)
Melanoma	2 (1)	2 (1)
Colon	3 (2)	3 (2)
Kidney	0 (0)	2 (1)
Other	2 (1)	4 (3)

Data are expressed as n (%). CMV, cytomegalovirus; PTLD, Post-Transplant Lymphoproliferative Disease.

**TABLE 7:** Non-adjusted and IPTW-weighted logistic regression for infection and new-onset neoplasms.

	OR [95% CI]	P value
Non-adjusted		
Infections that require hospitalization	1.18 [0.74–1.89]	0.49
CMV infection	0.39 [0.21–0.70]	0.002
BK infection	0.28 [0.10–0.77]	0.01
New onset neoplasms	1.72 [0.76–3.93]	0.20
IPTW adjusted		
Infections that require hospitalization	1.45 [0.92–2.33]	0.11
CMV infection	0.41 [0.23–0.72]	0.002
BK infection	0.31 [0.12–0.80]	0.02
New onset neoplasms	1.24 [0.48–3.21]	0.66

^a^ The Thymoglobulin group was considered the reference group. CMV, cytomegalovirus.

The incidence of new onset neoplasms was similar between both groups (6% vs. 10% for thymoglobulin and basiliximab groups, respectively, P = 0.21). Median time to neoplasm diagnosis was 73 [39–90] and 170 [95–201] months for thymoglobulin and basiliximab groups, respectively (P = 0.08). In this case, no significant association was identified between basiliximab and neoplasm development compared to thymoglobulin, either in the unadjusted [OR 1.72 (0.76–3.93), P = 0.20] or IPTW-weighted analysis [OR 1.24 (0.48–3.21), P = 0.66] (Tables 6, 7).

## Discussion

T-cell depleting agents (as thymoglobulin or alemtuzumab) and the IL2R blocker basiliximab have become the most frequently used induction agents in SPKT [5, 8, 13]. Nevertheless, information regarding post-transplant outcomes for each treatment remains controversial. Thus, in the present study we retrospectively compared post-transplant outcomes in a cohort of low immunological risk SPKT recipients after using either thymoglobulin or basiliximab as induction agents, focusing on long-term patient and grafts survival, as well as the incidence of post-transplant infections and neoplasms. Recipient, pancreas and kidney graft survival were similar among the two studied groups, as well as the incidence of graft rejection. Remarkably, basiliximab was not associated with a higher risk of pancreas and kidney graft rejection but significantly reduced the risk of CMV and BKV infection compared to thymoglobulin.

A multicenter randomized clinical trial demonstrating the benefit of induction therapy in SPKT was published in 2003 and ever since the use of induction therapy in pancreas transplantation has become almost ubiquitous [13, 14]. In this study, no differences were observed on 12-month graft survival between T-cell depleting agents or IL2R blockers. Nevertheless, in 2015, Kopp et al [15] reported a higher rate of pancreas rejection with IL2R blockers compared to Thymoglobulin in a long-term follow up study over 30 years in pancreas transplantation, although no differences in graft survival were observed. Since then, T-cell depleting agents have progressively gained relevance over IL2R blockers in the last decade, representing up to 80% of induction agent used in the USA [14]. Some studies have previously compared T-cell depleting agents and IL2R blockers as induction therapy in SPKT [9, 10, 16]. In 2011, Bazerbachi et al [9]. reported no differences for recipient and pancreas graft survival after 5 years, although basiliximab increased by 7-times the risk of pancreas TCMR at 1 year. Similar results were reported by Aziz et al [10] with a larger cohort of pancreas recipients. Remarkably, no increased risk of pancreas rejection was observed when only low immunological risk patients were considered (defined as cPRA <10%), although no information about pre-transplant DSAs was available. Our results are in line to those reported by Aziz et al, although with a longer follow up. Furthermore, in our study we considered as low immunological risk patients those with a cPRA <20% and no pre-transplant DSAs. The gap between cPRA cut off may be explained by the possibility to measure DSAs before transplant, which would allow to consider patients with a cPRA between 10% and 20% as low risk cases. Nevertheless, although no statistically significant differences in kidney or pancreas rejection rates were observed between thymoglobulin and basiliximab, our data showed a numerical trend toward higher rejection in the basiliximab group, as previously reported in other studies [17, 18]. Nevertheless, this tendency did not translate into inferior long-term graft or patient survival. This observation highlights the importance of careful recipient selection when considering basiliximab to avoid clinically relevant increases in rejection.

Thymoglobulin has also become the preferred induction agent in DCD pancreas transplantation due to a theoretically increased risk of rejection because higher severity of ischemia-reperfusion injury compared to Donors After Brain Death (DBD) [19]. Different studies have demonstrated that pancreas graft survival, patient survival, and rates of acute rejection are equivalent between DCD and DBD pancreas transplants, although thymoglobulin is the most frequent induction agent [19–21]. In our study, the proportion of DCD donors was higher in the thymoglobulin group, although CIT for both pancreas and kidney grafts were slightly longer in the basiliximab group. These findings suggest that basiliximab may also be effective in settings involving prolonged CIT. However, the higher incidence of DCD donors in the thymoglobulin group limits the ability to draw definitive conclusions regarding the efficacy of basiliximab in DCD transplants. This underscores the need for individualized selection of induction therapy based on the specific donor–recipient profile.

Post-transplant infections and neoplasms are two of the most important complications associated with T-cell depleting agents because their profound immunosuppressant effect [11, 12]. In our cohort, thymoglobulin was associated with an increased risk of CMV infection (up to 60%) compared to basiliximab. Noticeably, this observation persisted even when adjusting for confounding factors and considering that a higher number of recipients with CMV mismatch were present in the basiliximab group. These data are in line to those reported previously [10]. Similar to CMV, thymoglobulin increased the risk of BK virus infection up to 70% compared to basiliximab, a finding that has been previously suggested but no solidly demonstrated in those studies comparing thymoglobulin and basiliximab in SPKT [9, 10, 16]. No differences in the risk of post-transplant neoplasms were observed between the two study groups; however, neoplasms tended to occur earlier in the thymoglobulin group, suggesting a potential adverse effect associated with thymoglobulin use. Moreover, it has to be considered that, according to our center policy, induction with basiliximab was changed to thymoglobulin after 2013, thus conferring to the thymoglobulin cohort a shorter follow up that can falsely reduce the incidence of new onset neoplasms.

The results of our study reinforce some of the recommendations from the First World Consensus Conference on Pancreas Transplantation, particularly those regarding the impact of non-depleting agents on patient and graft outcomes [5]. In addition, the observed tendency toward earlier neoplasm development may support concerns about a higher risk of oncologic complications with depleting agents, an issue also highlighted in that Consensus.

Although our study focused on thymoglobulin and basiliximab as induction agents, these findings may also be relevant when evaluating alemtuzumab, another T-cell depleting agent used in pancreas transplantation. Previous studies in pancreas transplantation have suggested that alemtuzumab achieve comparable graft and recipient outcomes to thymoglobulin [22–24]. In this context, our findings indicating that basiliximab provides equivalent long-term outcomes to thymoglobulin, with a lower incidence of CMV and BK virus infection, raise the possibility that non-depleting IL2R blockers might offer a safer alternative even in comparison to alemtuzumab, at least for carefully selected low-risk recipients. This hypothesis has been recently addressed in a retrospective study performed by Swaab et al. [25]. They reported similar short-term graft outcomes between IL2R blockers and alemtuzumab induction in a small cohort of SPKT recipients, in line with previous studies [10]. Future studies directly comparing basiliximab, thymoglobulin, and alemtuzumab could therefore provide valuable guidance for tailoring induction therapy according to individual immunologic risk and infection susceptibility.

An additional consideration arising from our results is the potential role of “no induction” protocols in selected SPK recipients. Our cohort included exclusively SPK recipients, a population known to have lower immunologic risk compared with other pancreas transplant modalities (pancreas transplant alone and pancreas after kidney), and further restricted to low-risk immunologic profiles [17, 18, 26]. Given this context and the outcomes observed, it is conceivable that similar results could be achieved in carefully selected low-risk SPK recipients even without induction therapy, as has been suggested in prior studies [27, 28]. Therefore, future prospective studies are warranted to evaluate this strategy and to identify the patient characteristics that may allow safe omission of induction therapy.

Our study has the inherent limitations of a single-center, retrospective design. In addition, the two induction treatments were administered during different time periods, so a potential year effect cannot be entirely excluded. The choice of induction therapy followed institutional policy, with a transition from basiliximab to thymoglobulin after 2013. Nevertheless, the single-center setting ensured a homogeneous cohort, particularly in terms of SPKT management and treatment protocols, thereby reducing the risk of bias. Furthermore, our analysis accounted for improvements in pancreas transplantation observed since 2008, which also helps minimize bias related to the different administration periods of thymoglobulin and basiliximab.

With a follow-up period spanning 20 years, our findings add valuable long-term data on induction therapy in SPKT. Specifically, our results indicate that in recipients with low immunological risk, basiliximab offers comparable patient and graft survival outcomes to thymoglobulin, while being associated with a lower incidence of opportunistic infections post-transplantation. Randomized controlled trials are necessary to draw definitive conclusions about the optimal induction therapy for SPKT.

## Data Availability

The raw data supporting the conclusions of this article will be made available by the authors, without undue reservation.

## Abbreviations

ABMR, Antibody-Mediated Rejection; CIT, Cold Ischemia Time; CMV, Cytomegalovirus; cPRA, Calculated Panel Reactive Antibody; DBD, Donor After Brain Death; DCD, Donor after Circulatory Death; DGF, Delayed Graft Function; DM, Diabetes Mellitus; DSA, Donor Specific Antibodies; ESKD, End-Stage Kidney Disease; IPTW, Inverse Probability of Treatment Weighting; IQR, Interquartile Range; MFI, Mean Fluorescence Intensity; PDRI, Pancreas Donor Risk Index; PTLD, Post-Transplant Lymphoprolipherative Disorders; SD, Standard Deviation; SPKT, Simultaneous Pancreas-Kidney Transplantation; TCMR, T Cell-Mediated Rejection.
