# Supplementary material for: Impact of Induction Therapy in Low Immunological Risk Simultaneous Pancreas-Kidney Transplantation
Source: Transpl Int. 2025 Sep 30;38:15263. doi: 10.3389/ti.2025.15263 (PMC12518172; doi:10.3389/ti.2025.15263)
Supplement: Supplementary file 1 [file DataSheet1.docx]

SUPPLEMENTARY MATERIAL

Table S1– Standardized differences before and after IPTW adjustment

|  | Before IPTW adjustment | After IPTW adjustment |
| --- | --- | --- |
| Age at SPKT | -0.054 | 0.138 |
| Diabetes Mellitus duration at SPKT | 0.138 | 0.143 |
| Dialysis duration | -0.385 | -0.118 |
| Waiting list duration at SPKT | -0.414 | -0.067 |
| Smoking habit | 0.147 | -0.188 |
| Transplant era (after 2008) | -0.283 | 0.184 |
| High risk of CMV infection | -0.362 | -0.123 |
| Total HLA mismatches | -0.139 | 0.059 |
| cPRA pre-transplant | -0.023 | -0.121 |
| Maintenance immunosuppression |  |  |
| FK + MMF | -0.102 | -0.048 |
| FK + mTORi | 0.193 | 0.147 |
| CsA + MPS | -0.082 | -0.078 |
| Prednisone withdrawal | -0.131 | -0.021 |
| PDRI risk | -0.142 | 0.093 |
| Kidney CIT | -0.787 | -0.098 |

Table S2 – Recipient Cause of Death

|  | Thymoglobulin (n = 12) | Basiliximab (n = 19) | P value |
| --- | --- | --- | --- |
| Cardiovascular Disease | 1 (8) | 2 (11) | 0.55 |
| Infection | 6 (50) | 7 (37) |  |
| Neoplasm | 4 (34) | 5 (26) |  |
| Other | 1 (8) | 4 (21) |  |
| Unknown | 0 (0) | 1 (5) |  |

Data are presented as n (%) among the total recipient deaths in each group.


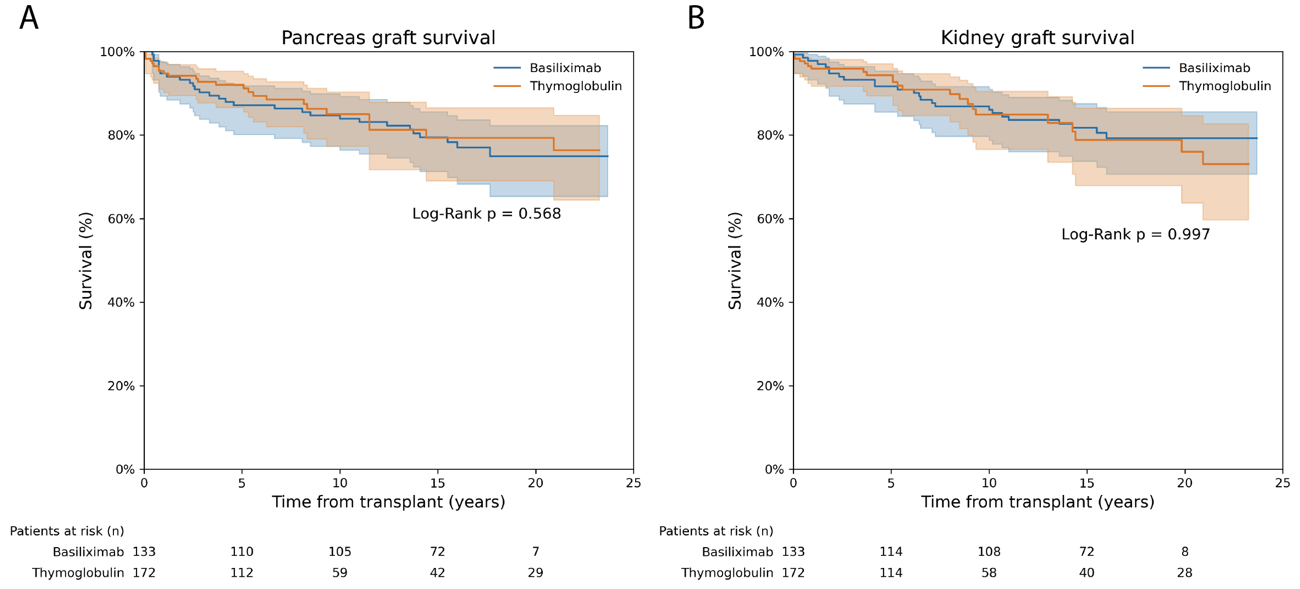


Figure S1 – Overall survival for kidney and pancreas grafts. A, Overall pancreas graft survival for patients treated with thymoglobulin and basiliximab. B, Overall kidney graft survival for patients treated with thymoglobulin and basiliximab.
